# Extracorporeal Blood Purification Techniques in Septic Shock: Operating Principles, Supporting Evidence and Clinical Applications

**DOI:** 10.3390/jcm15218619

**Published:** 2026-11-05

**Authors:** Marco Bonsano, Barbara Iacoviello, Giorgio Berlot

**Affiliations:** Department of Anesthesia, Intensive Care Medicine and Pain Therapy, Azienda Sanitaria Universitaria Giuliano-Isontina (ASUGI), 34149 Trieste, Italy; marco.bonsano@gmail.com (M.B.); b.iacoviello@yahoo.it (B.I.)

**Keywords:** septic shock, blood purification, hemoadsorption, hemofiltration, therapeutic plasma exchange

## Abstract

Several extracorporeal blood purification techniques are used to treat septic shock and other conditions characterized by the release of inflammatory mediators and bacterial toxins, notably lipopolysaccharide (endotoxin), which account for the cardiovascular, coagulation and metabolic derangements. Each technique has advantages and drawbacks, including, for most devices, the need for circuit anticoagulation and the possible removal of antibiotics. High-quality randomized trials remain scarce, and their heterogeneity in populations, techniques, dose and timing, together with the enrollment of biologically unselected patients, limits firm conclusions. In this narrative review, we describe the available techniques, critically appraise the clinical evidence for each modality against the 2026 Surviving Sepsis Campaign guidelines, and outline the phenotype-guided direction of ongoing research. The 2026 Surviving Sepsis Campaign guidelines suggest against the use of blood purification techniques in adults with sepsis or septic shock (conditional recommendation, very low certainty of evidence) and, specifically, against polymyxin-B hemoperfusion (conditional recommendation, low certainty of evidence). In the phenotype-enriched Tigris trial, polymyxin-B hemoadsorption showed a high posterior probability of mortality benefit in endotoxic septic shock; given the small sample, the imprecise primary estimate and the informative prior, this promising finding requires independent confirmation. Outside clinical trials, routine blood purification is not supported.

## 1. Introduction

Septic shock remains a leading cause of death in intensive care despite significant advances in antimicrobial therapy, hemodynamic optimization, and organ support [1,2]. Its pathophysiology is rooted in a dysregulated host response to infection, driven by the excessive release of molecular patterns associated either with the pathogen itself or with the tissue damage it causes. These pathogen- and damage-associated molecular patterns (PAMPs and DAMPs, respectively) cause endothelial dysfunction, microcirculatory failure, and progressive multi-organ dysfunction [3,4]. Septic shock is defined according to Sepsis-3 as a subset of sepsis in which profound circulatory, cellular and metabolic abnormalities substantially increase mortality, and is clinically identified by the need for vasopressors to maintain a mean arterial pressure of at least 65 mmHg and by a serum lactate level above 2 mmol/L despite adequate volume resuscitation [1]. Because many trials and meta-analyses of blood purification enrolled broader populations with severe infection or sepsis, the population of each source is specified throughout this review whenever it differs from septic shock. Within this framework, extracorporeal blood purification (EBP) techniques have been introduced as adjunctive therapies aimed at reducing the circulating burden of inflammatory mediators, endotoxin, and other pathogenic molecules when conventional treatment fails to restore immune and hemodynamic stability [5,6,7].

EBP includes a variety of modalities: hemofiltration, hemoadsorption, plasma exchange, and coupled plasma filtration–adsorption. Each relies on different physicochemical mechanisms such as diffusion, convection, and adsorption [8,9,10]. Advances in sorbent technology and membrane design have improved biocompatibility and efficiency, renewing interest in their use in this setting [6]. Adsorptive techniques, in particular, allow direct or plasma-mediated contact between blood and highly porous polymer matrices, facilitating the removal of both pro- and anti-inflammatory mediators and, in some cases, bacterial components [7,8,9]. Experimental and early clinical data suggest that the controlled reduction of circulating mediators may help restore immune homeostasis and stabilize hemodynamics, although a survival benefit remains unproven [4,5]. Reducing circulating mediator concentrations is a plausible therapeutic goal, because lowering them below a critical threshold or blunting their peak levels may limit the resulting organ damage [4]. Lowering plasma mediator concentrations may also increase the lymphatic drainage of mediators from tissues into the bloodstream, where they can be removed extracorporeally (the “mediator delivery hypothesis”), and restore the chemokine gradient from plasma to the infected tissue, thereby favoring leukocyte trafficking toward the site of infection [10]. In addition, extracorporeal contact with circulating leukocytes can itself modulate their function, as shown for granulocyte and monocyte adsorptive apheresis in experimental sepsis [11].

These effects probably coexist and vary over the course of treatment, together contributing to the therapeutic effect of EBP [8,10]. The efficacy of different EBP strategies depends on the interaction between the target mediator(s) and the device used. Removal of a given mediator is limited primarily by its molecular weight (MW) and by its hydrophilic or hydrophobic properties. Molecules exceeding a membrane’s MW cut-off are not cleared by convection and require alternative devices with different characteristics, such as pore size for convective clearance or adsorptive surfaces for hemoadsorption (HA).

The 2026 Surviving Sepsis Campaign (SSC) guidelines suggest against the use of blood purification techniques, including hemoperfusion, high-dose hemofiltration or plasma exchange, in adults with sepsis or septic shock (conditional recommendation, very low certainty of evidence) and, specifically, against polymyxin-B hemoperfusion (conditional recommendation, low certainty of evidence): the panel was uncertain of their effect on mortality and judged that, in the absence of clear benefit, their substantial resource requirements and potential impact on health equity did not justify their use [12]. Consistent with this, the 30th Acute Disease Quality Initiative (ADQI) consensus regards hemoadsorption as a novel and experimental intervention with limited data and a large research agenda, deliberately refrains from issuing practice recommendations and identifies possible target phenotypes, derived from endotoxin-based studies, for future randomized trials [7].

This narrative review aims to provide a comprehensive summary of the current evidence and clinical landscape of EBP techniques in septic shock. It covers the operating principles of each technique, the physiological and molecular rationale for its use, and the latest clinical evidence for and against its use in practice, framed against the conclusions of the 2026 SSC guidelines [12]. Compared with our previous review of blood purification techniques [13], the 30th ADQI consensus on hemoadsorption [7] and other recent reviews [6,14], the present work adds three elements: (i) a modality-by-modality appraisal against the 2026 SSC recommendations, including how these changed from the 2021 guideline [2,12]; (ii) the incorporation of the recently published Tigris trial, the first phase 3 trial to test prospectively the endotoxin-defined subgroup identified post hoc in EUPHRATES (endotoxin activity 0.60–0.89 with multiple organ dysfunction) [15,16]; and (iii) an explicit phenotype- and timing-based framework, which extends the patient-selection and timing considerations of our previous review [13] and links the operating principle of each technique to the biological target it can plausibly modify. This framework is intended as a hypothesis-generating guide to patient selection for future trials: apart from the endotoxin-defined population tested prospectively in Tigris [16], the phenotypes it describes have not been validated in clinical trials.

**Classification and operating principles**

EBP techniques fall into two broad families: blood-processing techniques, in which whole blood contacts a membrane or sorbent, and plasma-processing techniques, in which plasma is first separated and then either treated and returned, as in coupled plasma filtration–adsorption, or discarded and replaced, as in plasma exchange. The mediators a given device can remove are determined mainly by the target molecule’s molecular weight (MW) and hydrophilicity: small and middle-MW solutes such as inflammatory cytokines are cleared by convection across standard membranes or by adsorption, larger molecules require high-cut-off membranes (in an experimental swine model of lethal Staphylococcus aureus sepsis, continuous arteriovenous hemofiltration with a 100 kDa filter nearly doubled survival time compared with a 50 kDa filter, possibly through greater removal of higher-molecular-weight mediators [17]), and species confined to the intravascular compartment or bound to plasma proteins are addressed by plasma exchange [8,10,13]. Endotoxin does not behave like a conventional middle-MW solute: once in the circulation it is rapidly sequestered by complement, lipopolysaccharide-binding protein and high-density lipoprotein (HDL) [18], and, as a large, negatively charged molecule, it is removed mainly by adsorption, either to immobilized polymyxin B or to positively charged surfaces such as the polyethyleneimine-coated oXiris^®^ membrane [8,18].

Two non-mutually-exclusive paradigms justify these interventions (Figure 1). The first, the peak-concentration hypothesis, holds that transiently lowering the peak burden of circulating mediators below an injurious threshold limits organ damage, a rationale rooted in the continuous removal of middle-molecular-weight solutes [4]. The second frames the goal not as maximal removal but as the restoration of immune homeostasis, attenuating the pro-inflammatory surge while avoiding excessive depletion of mediators needed for pathogen control and repair [8,10]. These paradigms carry opposite risks: a purely subtractive, non-selective strategy that is beneficial during a hyper-inflammatory peak may be harmful once the host has transitioned to an immunosuppressed, hypo-inflammatory state. Effective therapy therefore depends less on adsorptive capacity per se than on matching the removal target, and its timing, to the patient’s prevailing immune phenotype.

Hemofiltration (HF) removes water and hydrophilic solutes, including inflammatory mediators up to a membrane cut-off of ~50 kDa, by convection (Figure 2); its intensity is governed by the ultrafiltration rate (Qf), together with blood flow (Qb), membrane permeability and surface area [8,10,13]. Mediator clearance has been pushed further by high-cut-off membranes (at the cost of albumin loss), by dual-filter (“cascade”) circuits, and above all by higher Qf, on which HF is conventionally graded: standard-volume continuous veno-venous hemofiltration (CVVH, 25–35 mL/kg/h), high-volume (35–60), very-high-volume (>60) and pulse high-volume regimens [19,20,21]. This dose classification, used to appraise the trials below, is applied consistently throughout this review.

Hemoadsorption removes circulating molecules by binding them to a sorbent through weak ionic and hydrophobic interactions; performance depends on Qb and on the affinity and capacity of the sorbent (Figure 3). Contemporary devices use biocompatible polymer resins and porous synthetic materials with very large surface-area-to-mass ratios, configured as beads, fibers or discs; individual cartridges differ in sorbent chemistry, target and surface area (Supplementary Table S4) [22]. Some newer sorbents also bind whole pathogens (bacteria and viruses) together with pathogen- and damage-associated molecular patterns.

Therapeutic plasma exchange (TPE) removes plasma either non-selectively, with donor-plasma or albumin replacement, or selectively (plasmapheresis), targeting high-MW pathogenic species; when fresh-frozen plasma is the replacement fluid, it also repletes protective factors depleted in sepsis (Figure 4) [23].

Regardless of family, these techniques share a decline in clearance as the sorbent saturates or the membrane fouls, and the need for large-bore vascular access and, in most configurations, circuit anticoagulation (typically unfractionated heparin or regional citrate). The need for anticoagulation and the choice of agent depend on the device and circuit, on whether the device is integrated into concurrent continuous renal replacement therapy (CRRT), which is possible for most blood-processing devices (Supplementary Table S3), and on the patient’s bleeding and thrombotic risk [13].

## 2. Methods

This narrative review offers an interpretative appraisal of the current evidence on EBP in septic shock. Although the search and the selection of evidence are reported transparently, this review does not adopt the full methodological structure of a systematic review (registered protocol, duplicate screening, formal risk-of-bias assessment and quantitative synthesis), which lies outside its scope; the eligibility criteria below describe how the evidence discussed was selected. To improve transparency and limit selection bias, the literature was identified through a structured search of MEDLINE (via PubMed) and Embase using a predefined Boolean strategy combining terms for sepsis/septic shock with terms for each EBP modality (full search strings in Supplementary Material S2). To update the established evidence base, the search was restricted to 1 January 2022–5 August 2025; landmark trials, pivotal randomized controlled trials (RCTs), systematic reviews, meta-analyses and consensus documents published before this window were incorporated through hand-searching of retrieved reference lists and of the 2026 SSC guideline [12]. The MEDLINE search was updated on 23 October 2026 to capture evidence published after the original window, including the 2026 SSC guidelines [12], the Tigris trial [16], two further RCTs of hemoadsorption in septic shock [25,26] and an updated network meta-analysis [27]. The update also identified a pilot RCT of HA380 hemoadsorption in sepsis-associated acute kidney injury [28], an umbrella review of systematic reviews [29] and a position statement on hemoadsorption [30]; the records retrieved and screened in MEDLINE are reported in Supplementary Material S2. The update was restricted to MEDLINE because its purpose was targeted rather than exhaustive: to identify the major RCTs, guidelines and evidence syntheses published after the original search window, which are consistently indexed in MEDLINE; the reference lists of the newly identified records were also hand-searched. Because Embase was not searched for this period, studies indexed only in Embase may have been missed, and we acknowledge this as a limitation of the literature coverage of this review. The following eligibility criteria were applied. We included RCTs, systematic reviews with or without meta-analysis and guideline or consensus documents evaluating an EBP modality in adults with sepsis or septic shock. Because this review focuses on septic shock, studies restricted to septic shock were prioritized; studies enrolling broader populations (severe infection, or sepsis without shock) were eligible when they formed part of the pooled syntheses on which current recommendations rest, and their population is identified as such in the text and in Supplementary Table S1. Pediatric-only studies were excluded, except where they contributed to pooled estimates of the source reviews (e.g., the plasma-exchange review of Rimmer et al. [31]). Studies in COVID-19 were eligible only when patients had septic or vasoplegic shock and were identified as such. Studies of renal replacement therapy delivered for acute kidney injury (AKI) were excluded unless they compared a blood purification strategy (dose or device) in patients with sepsis. Conference abstracts were excluded. Animal, volunteer and non-English studies, and studies in other critically ill populations (e.g., the CYCOV trial in patients with COVID-19 receiving extracorporeal membrane oxygenation, or meta-analyses of CytoSorb^®^ across indications), were not used as evidence of efficacy in septic shock and are cited only for mechanism, device principles, safety signals or their sepsis subgroups, as indicated in the text. Records were de-duplicated in Rayyan. We prioritized RCTs, systematic reviews with meta-analysis, and guideline/consensus statements; observational data and case series were cited where they illustrate mechanism, feasibility or off-trial practice. Where the evidence base has been formally synthesized, we adopted the effect estimates, risk-of-bias judgments and GRADE certainty of the source reviews rather than re-deriving them: principally the network meta-analysis of Chen et al. [32] for hemoperfusion/hemadsorption and hemofiltration and the systematic review of Rimmer et al. [31] for plasma exchange, both of which underpin the 2026 SSC recommendations. The characteristics of the individual RCTs discussed are tabulated in Supplementary Table S1.

**Evidence from clinical research**

Since the late 1980s, dozens of controlled trials have tested EBP in sepsis and septic shock, yet the evidence remains discordant: individual studies variously report benefit, no effect, or harm [32,33]. The most complete appraisal of this body of work is the network meta-analysis of Chen et al. [32], which pooled 60 RCTs and 4595 critically ill adults with severe infection, sepsis or septic shock (trials published 1999–2022) across sixteen modalities, and which, together with the plasma-exchange systematic review of Rimmer et al. [31], underpins the 2026 SSC recommendations [12]. A subsequent Bayesian network meta-analysis restricted to RCTs in severe septic shock (31 RCTs, 2678 patients, 15 treatments), based on fixed-effect summary estimates, found lower mortality only with polymyxin-B and HA330, and higher mortality with convective treatment at any dose [27]. An umbrella review of 42 systematic reviews and meta-analyses reached a similar conclusion: pooled estimates generally favor EBP, with the most consistent direction of effect for polymyxin-B hemoperfusion, but the certainty of evidence for mortality is low and most of the underlying reviews are of low or critically low methodological quality [29]. Two features of that appraisal frame the discussion that follows. First, the certainty of evidence is low or very low for almost every modality: in Chen’s GRADE assessment fourteen of sixteen interventions were rated very low, one low and one moderate, and only 19 of 60 trials were at low risk of bias (15 were at high risk). Second, the discordance is largely explained by four recurring confounders, which we adopt as the analytic lens for each technique: (a) heterogeneous populations and underlying pathologies; (b) variable co-interventions (antimicrobial adequacy, source control, vasopressor and nutritional strategy); (c) differences in the EBP prescription itself (timing, dose, session duration); and (d) inconsistent outcome definitions (mortality vs. biomarker or vasopressor surrogates). Throughout, we distinguish physiological or surrogate outcomes (mediator clearance, vasopressor dose, sequential organ failure assessment [SOFA] score trajectory) from patient-important clinical outcomes; improvements in the former are not interpreted as evidence of survival benefit. Critically, only 3 of the 60 trials enrolled patients by a biological criterion (endotoxin activity assay > 0.6), so most trials applied mechanism-specific therapies to biologically unselected patients, which is the single most important reason why the aggregate signal is weak.

Accordingly, the 2026 SSC issued two recommendations for adults with sepsis or septic shock [12]. First, “we suggest against using blood purification techniques”, including hemoperfusion, high-dose hemofiltration or plasma exchange (conditional recommendation, very low certainty of evidence). Second, “we suggest against using polymyxin B hemoperfusion” (conditional recommendation, low certainty of evidence). By modality, therefore: (i) hemofiltration—only high-dose hemofiltration is addressed, within the first recommendation (conditional, against, very low certainty); (ii) polymyxin-B hemoperfusion—a specific recommendation (conditional, against, low certainty); (iii) other hemoperfusion/hemoadsorption devices (e.g., CytoSorb^®^, oXiris^®^, HA330, Seraph^®^ 100)—no device-specific recommendation, being covered by the first recommendation (conditional, against, very low certainty); and (iv) plasma exchange—no separate recommendation, being likewise included in the first recommendation, although the panel reported a pooled estimate for plasmapheresis (4 RCTs) in its evidence summary. Compared with the 2021 guideline, which suggested against polymyxin-B hemoperfusion (weak recommendation, low quality of evidence) but judged the evidence insufficient to make a recommendation on other blood purification techniques [2], the 2026 update retains the recommendation against polymyxin-B hemoperfusion and extends a conditional recommendation against use to blood purification as a whole, on the basis of updated systematic reviews of 41 RCTs of hemoperfusion/hemadsorption, 26 of hemofiltration and 4 of plasmapheresis [12]. The modality-level estimates that follow, together with the corresponding SSC recommendation, are summarized in Table 1.

**Hemofiltration**

Early studies suggested a dose–response relationship between Qf and survival [20,34], and high-volume hemofiltration improved hemodynamics in septic shock [35], but this hypothesis has not survived controlled testing. The IVOIRE trial, comparing very-high-volume (70 mL/kg/h, according to the classification adopted here) with standard-volume (35 mL/kg/h) hemofiltration in septic shock with AKI, found no clinical benefit [36], while very-high-volume hemofiltration (120 mL/kg/h) with the Cascade system, designed to increase mediator clearance while limiting albumin loss [21], did not reduce catecholamine requirements or mortality compared with usual care in a randomized trial of 60 patients with septic shock [37]; high cut-off membranes increase cytokine sieving and mass removal but, in critically ill patients with AKI, did not lower plasma cytokine levels more than standard filters [38]. Elevated Qf additionally accelerates antibiotic clearance, risking subtherapeutic exposure [39]. Many of these trials enrolled patients with sepsis-associated AKI or mixed critically ill populations rather than septic shock alone (Supplementary Table S1). These findings are confirmed quantitatively: in Chen’s network meta-analysis, no hemofiltration dose reduced mortality compared with standard care, with relative risks (RR) of 0.86 (95% CI 0.61–1.23) for standard-volume CVVH, 0.67 (0.41–1.10) for high-volume, 0.71 (0.43–1.15) for very-high-volume and 0.50 (0.05–5.01) for pulse high-volume hemofiltration, all at very low certainty [32]. The updated 2026 SSC pooled estimate for hemofiltration (26 RCTs) shows a short-term (28–30 day) signal (RR 0.61; 95% CI 0.61–0.90 as printed in the guideline, low certainty; because the point estimate coincides with the lower confidence limit, this figure most likely contains a typographical error in the source and should be interpreted with caution) that does not persist at longer follow-up (RR 0.93; 95% CI 0.78–1.11, very low certainty, across 5 RCTs) [12]. In sum, convective-dose escalation confers no survival benefit; the residual short-term signal is of low certainty, downgraded for risk of bias and inconsistency, and the earliest blood purification trials, including some with the largest effects, are over 20 years old and reflect sepsis care inconsistent with current standards [12].

**Hemoadsorption**

***Polymyxin-B hemoperfusion (Toraymyxin^®^)***

Polymyxin-B hemoperfusion (PbH) is the most extensively studied adsorptive technique, and its trials are the clearest illustration of the phenotype problem. The small EUPHAS trial (64 patients) reported improved hemodynamics and organ dysfunction and lower 28-day mortality, a secondary endpoint [40], whereas the larger ABDOMIX trial in peritonitis-associated septic shock showed a trend toward higher mortality [41]; an ancillary analysis of ABDOMIX explored its effect on the inflammatory response [42]. The pivotal EUPHRATES trial, which enrolled patients with elevated endotoxin activity (endotoxin activity assay [EAA] ≥0.60), was neutral overall [43], but a post hoc analysis found a survival benefit confined to the subgroup with intermediate endotoxin activity (EAA 0.60–0.89) and multi-organ failure [15]. Higher endotoxin burden has itself been associated with mortality in peritonitis-induced shock [44]. This endotoxin-defined subgroup was subsequently tested prospectively in the Tigris trial (NCT03901807; see below) [16]. Quantitatively, Chen’s network meta-analysis assigns PbH a pooled mortality RR of 0.70 (95% CI 0.58–0.86) but at very low certainty; the estimate fails sensitivity analysis once high-risk-of-bias trials are excluded, trial-sequential analysis was reported as true-positive with a nearly sufficient sample size, and the effect was larger in Asian populations and older studies [32]. The updated 2026 SSC pooled short-term estimate for hemoperfusion/hemadsorption as a whole (41 RCTs, all devices, not polymyxin-B alone) is RR 0.83 (95% CI 0.71–0.98, low certainty), again not durable at longer follow-up (RR 1.11; 95% CI 0.94–1.30, low certainty, across 4 RCTs), and the panel suggested against polymyxin-B hemoperfusion (conditional recommendation, low certainty) [12]. Both meta-analyses [32,33] locate the apparent benefit in older trials conducted in Asia, whereas trials at low risk of bias show no mortality difference [32,33].

The Tigris trial has since provided the first phase 3 test of this phenotype-enriched hypothesis [16]. In this open-label, Bayesian RCT conducted in 19 US hospitals, adults with septic shock, multiple organ dysfunction and an endotoxin activity of 0.60–0.89 units were randomized 2:1 to two sessions of polymyxin-B hemoadsorption plus standard care or standard care alone; of 14,890 patients screened, 157 were enrolled (106 vs. 51). Twenty-eight-day mortality was 39% (41/106) with polymyxin-B and 45% (23/51) with standard care (Acute Physiology and Chronic Health Evaluation [APACHE] II-adjusted odds ratio 0.67, 95% credible interval 0.39–1.08), corresponding to a posterior probability of benefit of 95.3%; for 90-day mortality, the key secondary outcome, the posterior probability of benefit was 99.4% (odds ratio 0.54, 95% credible interval 0.32–0.87). Serious adverse events occurred in 30/100 (30%) versus 11/51 (22%) patients, two (2%) of which were judged treatment-related [16]. These results should be interpreted in light of the open-label design, the small sample, a 28-day credible interval that includes no effect, the very narrow eligibility (about 1% of screened patients were enrolled), industry sponsorship and the use of an informative prior derived from the EUPHRATES subgroup (weighted at 75%), so that the reported posterior probabilities do not rest on the Tigris data alone. In the trial’s own sensitivity analysis with an uninformative prior, the 28-day posterior probability of benefit fell to 79.7% (odds ratio 0.80, 95% credible interval 0.38–1.49), whereas the 90-day result was robust to the choice of prior (98.4%; odds ratio 0.50, 95% credible interval 0.23–0.94) [16]. Tigris was published concurrently with the 2026 SSC guidelines, whose evidence review predates its results; their recommendation against polymyxin-B hemoperfusion therefore does not reflect its results [12,16]. In sum, PbH is not supported for unselected septic shock; in an endotoxin-defined phenotype (endotoxin activity 0.60–0.89 with multiple organ dysfunction), Tigris provides the first prospective, phenotype-enriched evidence suggestive of a mortality benefit. Given the small sample, the imprecision of the 28-day estimate and its sensitivity to the informative prior, this evidence should be regarded as promising rather than as an established clinical benefit, and it requires confirmation in independent, adequately powered trials.

***Cytokine adsorption (CytoSorb^®^)***

CytoSorb^®^ is the modality whose widespread use is least supported by evidence. Case series, observational cohorts and a small pilot RCT [45] describe reductions in cytokine levels and vasopressor requirements and, in non-randomized comparisons, lower-than-expected mortality [45,46,47,48,49,50], but these are inconsistently reproduced [51,52] and have been questioned [53], and its capacity to remove interleukin-6 and TNF-α is itself contested (negative in critically ill patients [54]; positive only in an endotoxin-challenge volunteer model [55]). Large observational data are difficult to interpret because of case-mix and timing [56]; single-center series suggesting benefit from earlier initiation [49] or from a higher volume of blood processed [50] are hypothesis-generating, not confirmatory. Systematic reviews are correspondingly split—a meta-analysis of RCTs in critically ill patients (not restricted to sepsis) found higher mortality with CytoSorb^®^ (RR 1.24; 95% CI 1.04–1.49; low certainty) [57]; no mortality benefit was found by Becker et al. across mixed indications, including sepsis (RR 0.98; 95% CI 0.74–1.31) [58], or by Saldaña-Gastulo et al. [59]; Orban et al. found no effect on overall mortality (OR 0.95; 95% CI 0.58–1.56) and higher mortality with earlier initiation (OR 2.02; 95% CI 1.06–3.85) [60]; only Steindl et al., restricted to septic shock, reported lower in-hospital mortality (OR 0.64; 95% CI 0.42–0.97), from one RCT and eight observational studies, while cautioning that the underlying evidence was of low certainty [61]. Importantly, CytoSorb^®^ is the only modality in Chen’s network meta-analysis carrying moderate-certainty evidence, and its point estimate favors harm (mortality RR 1.39; 95% CI 0.97–1.98) [32]; the randomized CYCOV trial reported significantly higher 30-day mortality with CytoSorb^®^ during extracorporeal membrane oxygenation (ECMO) for COVID-19 [62]. In the most recent RCT, restricted to septic shock with hyperinflammation (31 patients, single center), early CytoSorb^®^ did not reduce the cumulative norepinephrine dose over 72 h (primary endpoint) or modify cytokine levels, and survival at 48 and 72 h was lower with hemoadsorption (64% vs. 100% and 57% vs. 94%), without a difference in intensive care unit (ICU) mortality [25]. In sum, no RCT demonstrates a survival benefit, the highest-certainty pooled estimate trends toward harm, and routine use in sepsis is not evidence-supported.

***Neutro-macroporous resin hemoperfusion (HA330/HA380)***

HA330 cartridges contain neutro-macroporous styrene–divinylbenzene resin beads that adsorb molecules of approximately 500 Da to 60 kDa, including cytokines (Supplementary Table S4) [22]. In Chen’s network meta-analysis, HA330 alone (2 RCTs) did not significantly reduce mortality (RR 0.61; 95% CI 0.35–1.09; very low certainty) [32]. The multicenter CLEANSE RCT, conducted in Thailand in septic shock requiring norepinephrine ≥0.2 µg/kg/min and stopped early for slow recruitment and funding constraints (128 of 206 planned patients), found no significant reduction in 28-day mortality with two 3 h HA330 sessions (44% vs. 58%; RR 0.76, 95% CI 0.54–1.07) and no differences in shock reversal, interleukin-6 levels or organ support-free days, while hypotension occurred during hemoadsorption in 46% of treated patients [26]. In a single-center pilot RCT of 76 patients with sepsis-associated persistent severe AKI requiring CRRT, adding the related HA380 cartridge to CVVH did not significantly reduce 30-day mortality (36.8% vs. 52.6%; *p* = 0.159), the trial being unpowered for this endpoint; a post hoc analysis suggested benefit confined to patients with urinary C-C motif chemokine ligand 14 (CCL14) above 1.3 ng/mL, a hypothesis-generating finding that again points toward biomarker-based enrichment [28]. In sum, a possible but imprecise signal from small and underpowered trials; HA330 and HA380 are not supported for routine use outside clinical trials.

***Adsorptive hemodiafilter (oXiris^®^)***

The oXiris^®^ membrane combines endotoxin and cytokine adsorption with CRRT [63]. Clinical evidence is dominated by small series and heterogeneous cohorts reporting improvements in physiological and surrogate endpoints (vasopressor requirements, lactate, endotoxin and interleukin-6 concentrations, SOFA score) and lower-than-predicted mortality, a comparison that cannot establish clinical benefit [64,65] (reviewed in [66]), with the attendant bias emphasized by Li et al. [67]. A meta-analysis by Wang et al. (14 studies: 4 RCTs and 10 observational) associated oXiris^®^ with lower 28-day mortality (OR 0.53; 95% CI 0.36–0.77) and shorter ICU stay, but found no difference in 90-day, ICU or hospital mortality and rated the certainty of all outcomes as low or very low [68], and a further meta-analysis of studies selected for interleukin-6 removal reported lower vasopressor requirements and SOFA scores during treatment, but did not pool mortality and could not establish a causal link with interleukin-6 removal [69]. Reductions in interleukin-6, vasopressor dose or SOFA score are surrogate outcomes and do not demonstrate a survival benefit. These signals must be weighed against the network-meta-analytic estimate, in which oXiris^®^ did not significantly reduce mortality (RR 0.72; 95% CI 0.29–1.78), based on a single small RCT, at very low certainty [32]. In sum, improvements in hemodynamic and inflammatory surrogate markers during CRRT have been reported, but no survival benefit has been demonstrated, and the certainty of evidence for mortality is very low.

***Pathogen-adsorbing filter (Seraph^®^ 100)***

The Seraph^®^ 100 filter consists of polyethylene beads with endpoint-attached heparin, which mimics heparan-sulfate receptors and binds a broad spectrum of pathogens and DAMPs [70,71]. Evidence is limited to mechanistic and feasibility reports—a first-in-human study in 15 hemodialysis patients with bloodstream infection showing that a single 4 h session was well tolerated, with a non-significant prolongation of blood-culture time to positivity across the filter [72], and a report of two chronically critically ill patients whose secondary viral bloodstream infections were successfully treated [70]. No RCT reports a mortality outcome in sepsis, and the device is not included in the mortality network meta-analysis. In sum, a biologically rational pathogen-reduction strategy at an early, feasibility stage of evidence; no efficacy claim is currently supportable.

***LPS-selective adsorbent (Efferon LPS^®^)***

A single multicenter RCT (Rey et al., 60 patients randomized 2:1, 58 analyzed) evaluated the LPS-selective mesoporous adsorbent Efferon LPS^®^ in intra-abdominal septic shock [73]. Shock resolution (primary endpoint) did not differ significantly (68% vs. 45%; *p* = 0.098), although survivors were weaned from vasopressors faster, SOFA scores fell more at 24 h and 3-day mortality was lower (13% vs. 40%); 28-day mortality did not differ (47% vs. 55%). The trial was small and conducted in a single country, and it is not included in the network meta-analysis. In sum, a physiological signal in a single small RCT whose primary endpoint was not met and with no 28-day survival difference; hypothesis-generating only.

**Coupled plasma filtration and adsorption (CPFA)**

CPFA couples plasma separation, sorbent adsorption and continuous veno-venous hemodiafiltration. Two Italian RCTs (COMPACT [74] and the high-dose COMPACT-2 [75]) were both stopped early: COMPACT for futility (hospital mortality 45.1% vs. 47.3%), with an exploratory per-protocol signal of benefit at higher treated plasma volumes that was compromised by frequent under-delivery of the intended dose, and COMPACT-2 because of a possible harmful effect, with early excess deaths and a dose–response relationship between treated plasma volume and mortality, most marked in patients without severe renal failure. The ROMPA trial, closed prematurely after the COMPACT-2 safety alert, was inconclusive owing to low power (28-day mortality 57.9% vs. 46.7%) [76]. Chen’s network meta-analysis concurs: CPFA mortality RR 1.20 (95% CI 0.82–1.76), very low certainty [32]. The meta-analysis of Snow et al. likewise found no mortality benefit with CPFA (OR 0.50; 95% CI 0.10–2.47; 2 RCTs) [77]. The persistent discrepancy with favorable single-center experiences most plausibly reflects the absence of standardized “rules of engagement” and the loss of single-center protocol fidelity in multicenter practice. In sum, consistent randomized evidence of no benefit (and a harm signal); CPFA has effectively left the routine armamentarium.

**Therapeutic plasma exchange (TPE)**

TPE is mechanistically distinct: it can both remove high-molecular-weight mediators (including activated complement) and, when fresh-frozen plasma is the replacement fluid, replete protective factors depleted in sepsis: ADAMTS-13, protein C, protein S and antithrombin [23,78]. A 40-patient RCT of high-volume (>3000 mL) TPE reported a non-significant numerical trend toward lower 28-day mortality, faster vasopressor withdrawal and greater lactate clearance, with repletion of antithrombin, protein C and ADAMTS-13 [79]; Stahl et al. additionally demonstrated restoration of the endothelial glycocalyx damaged in sepsis, relevant to capillary leak and leukocyte adhesion [24]. Systematic reviews are supportive: Hernandez et al. (3 RCTs and 1 cohort study) pooled OR 0.43 (95% CI 0.26–0.72) [80] and Kuklin et al. (systematic review of 20 studies in 937 adults with sepsis-induced organ dysfunction; meta-analysis of 5 RCTs and 6 matched cohort studies, 627 patients) RR 0.59 (95% CI 0.47–0.74) [81], with effects in both COVID-19 and non-COVID-19 sepsis (not significant with membrane plasma filtration), although Luo et al. found no benefit and prolonged ICU stay in a small cohort [82], and Zhang et al. noted benefit chiefly in adults [83]. The core estimates that anchor the 2026 SSC are those of Chen (plasma exchange RR 0.61; 95% CI 0.42–0.91, low certainty—the highest-rated protective signal of any modality) [32] and Rimmer et al. [31], whose systematic review of 4 RCTs (194 patients, all at unclear-to-high risk of bias) found no overall mortality reduction (RR 0.83; 95% CI 0.45–1.52) but a significant benefit in the adult subgroup (RR 0.63; 95% CI 0.42–0.96) [31]. The SSC panel’s own updated estimate for plasmapheresis (4 RCTs; RR 0.64, 95% CI 0.46–0.89; low certainty) is more favorable than the 2014 estimate of Rimmer et al., reflecting the panel’s updated trial set and analysis; no plasma-exchange-specific recommendation was issued; plasma exchange is included in the general recommendation against blood purification [12]. Trial-sequential analysis for TPE was reported as true-positive, although the authors noted that additional studies are needed to confirm the benefit [32].

Safety deserves the same attention as for the other modalities. The fresh-frozen plasma (FFP) that repletes protective factors also exposes patients to the risks of allogeneic plasma: compared with albumin, plasma replacement carries a higher risk of anaphylactoid and other immune reactions, including transfusion-related acute lung injury, and a risk of transfusion-transmitted infection [23]. Citrate, used for circuit anticoagulation and contained in FFP, makes symptomatic hypocalcemia more common, which can be prevented by prophylactic calcium administration, and its metabolism may cause metabolic alkalosis when plasma is the replacement fluid [23]. Potentially life-threatening complications, dominated by anaphylactoid reactions and severe hypotension, are reported in 1–2% of sessions in critically ill patients, and hypotension is more likely when crystalloid replaces more than 30% of the removed volume, which is relevant in patients already receiving high-dose vasopressors [23]. Finally, TPE effectively removes highly protein-bound drugs with a low volume of distribution, including some antimicrobials; the timing of administration relative to a session depends on the drug’s pharmacodynamics (aminoglycosides are best given before a session, whereas beta-lactams often require a supplementary dose afterwards), and therapeutic drug monitoring is advisable [23,84]. No adverse events were observed in the 40-patient RCT of Stahl et al. [79], but the available RCTs were too small to characterize these risks reliably.

Two mechanistic points deserve emphasis. First, sepsis activates the complement cascade through all three pathways, and the resulting anaphylatoxins contribute to endothelial injury and the amplification of inflammation [3]; the effect of blood purification on activated complement is, however, technique-dependent and incompletely characterized. TPE can remove high-molecular-weight activated complement fragments, consistent with its broader immunomodulatory profile [78]. By contrast, direct evidence that adsorptive or convective techniques meaningfully lower activated complement in septic patients is scarce; moreover, contact between blood and the extracorporeal membrane or sorbent can itself activate complement and the contact system as a feature of bioincompatibility, so the net effect of a given device is uncertain and may be bidirectional. In sum, TPE carries the most coherent and biologically rationalized positive signal, but at low certainty and from small, biased trials; it should be regarded as adjunctive and investigational pending the adequately powered EXCHANGE-2 RCT (NCT05726825), which plans to randomize 274 patients with early septic shock and high vasopressor requirements to FFP-based TPE or standard care, with 28-day mortality as primary endpoint, and was still recruiting at the time of revision (October 2026) [85].

## 3. Discussion

The appraisal above yields a consistent message. Across roughly sixty randomized trials, no EBP modality has demonstrated a durable, low-risk-of-bias reduction in mortality in unselected septic shock; the only positive pooled signals—polymyxin-B hemoperfusion and plasma exchange—are of low or very-low certainty as graded by the source syntheses, and the single moderate-certainty estimate, for CytoSorb^®^, points toward harm [12,32]. Tigris, published concurrently with the 2026 SSC guidelines, is the first phase 3 trial to test prospectively the endotoxin-defined subgroup identified in EUPHRATES and reported a high posterior probability of mortality benefit; however, the 28-day estimate was imprecise and partly dependent on the informative prior, so these promising findings require independent confirmation, and they inform the use of polymyxin-B in endotoxic septic shock, not EBP in unselected septic shock [16]. The two most recent RCTs of cytokine-adsorbing devices in septic shock, with CytoSorb^®^ and HA330, showed no benefit on their primary endpoints [25,26]. Rather than re-examining these estimates, we consider here what they imply for practice and for the design of future trials.

The dominant lesson is biological non-selection. The therapies with the most plausible mechanism—endotoxin-directed hemoperfusion and mediator- and factor-directed plasma exchange—are precisely those whose only promising signals arise in phenotype-defined subgroups: the survival benefit of polymyxin-B hemoperfusion was confined to patients with elevated endotoxin activity and multi-organ failure [15], a post hoc finding since tested prospectively in Tigris, with promising but not yet confirmed results [16], and that of plasma exchange to adults, in an exploratory subgroup analysis [31]. This reframes the central question from “does blood purification work?” to “in whom, initiated when, and judged by which endpoint?” Analogous biomarker-guided logic recurs across modalities: interleukin-6 has been proposed as a surrogate for the cytokine burden most likely to respond to adsorptive membranes [69], and the depletion of ADAMTS-13, protein C and antithrombin that plasma exchange corrects [79] makes sepsis-associated coagulopathy a rational target for this technique. Underlying all of these is the distinction between hyper- and hypo-inflammatory sepsis endotypes: a non-selective subtractive intervention that benefits a patient at the inflammatory peak may harm one who has already progressed to immunoparalysis, which makes “when to treat” inseparable from “whom to treat”. This is the rationale for the sequential extracorporeal therapy (SET) concept [9], in which the modality and its timing are matched to the phase of the septic cascade rather than applied uniformly. These levels of evidence should, however, be kept distinct. Only one biomarker-defined population, septic shock with an endotoxin activity of 0.60–0.89 and multiple organ dysfunction, has been evaluated prospectively in a phase 3 trial, and only for polymyxin-B hemoperfusion [16]; the benefit of plasma exchange in adults [31] and the possible benefit of HA380 in patients with elevated urinary CCL14 [28] derive from exploratory subgroup or post hoc analyses; and the interleukin-6-defined, coagulopathic and hyper- or hypo-inflammatory phenotypes proposed for adsorptive membranes, plasma exchange and sequential therapy have not yet been validated as treatment-selection criteria in clinical trials. Accordingly, the phenotype-guided framework outlined here cannot be extrapolated from polymyxin-B hemoperfusion to other EBP modalities, for which it remains hypothesis-generating. Translating this into evidence requires trial architectures built for enrichment rather than breadth—adaptive, biomarker-stratified, Bayesian designs of the kind now advocated for sepsis research [86]—and the field’s most recent and ongoing trials are accordingly phenotype-enriched: the endotoxin-targeted Tigris trial for polymyxin-B hemoadsorption, now reported [16], and the ongoing EXCHANGE-2 trial [85] for plasma exchange. Other registered RCTs of adsorptive techniques include the OASES trial of oXiris^®^ in abdominal septic shock requiring CRRT (NCT06504316), which uses a hemodynamic primary endpoint, and the EABPSS trial of adaptive plasma filtration–adsorption in septic shock (NCT06692036; 276 patients), which uses 90-day mortality [87].

Pending further trials, and given that the 2026 SSC guidelines suggest against its use [12], any decision to consider EBP, preferably within a clinical trial or prospective registry, should at least take into account three variables: (a) the underlying disease trajectory, since these and other advanced therapies are futile in patients with terminal illness or very short life expectancy; (b) early, aggressive source control, because EBP complements rather than replaces antimicrobials and surgery; and (c) timing, as earlier initiation has been associated with better outcomes in observational data [49], although a meta-analysis of CytoSorb^®^ found higher mortality with earlier initiation [60], so the optimal timing remains undefined. Together with an explicit treatment target, these considerations may help prevent the use of EBP as a reflex response to refractory shock, but they do not substitute for evidence of clinical efficacy.

Three practical uncertainties persist even where EBP is considered. First, endpoint definition: survival alone is an unreliable signal given the dominant influence of source control and antimicrobial adequacy, so biochemical and hemodynamic surrogates (lactate clearance, vasopressor reduction, SOFA trajectory) and standardized composite endpoints such as vasopressor- and ventilator-free days are useful complementary endpoints, as the ADQI-30 consensus advocates, provided that they are not interpreted as evidence of survival benefit [7]. Second, undesired effects: beyond catheter- and anticoagulation-related risks, all techniques may cause hypothermia, complement activation at the blood–membrane interface, and unintended depletion of beneficial mediators, while antimicrobial adsorption warrants therapeutic drug monitoring or dose adjustment [14,88]. Third, appropriateness of timing and dose: as with antimicrobials, earlier intervention is plausible but unproven [49,60]; both under- and over-treatment may be harmful, and a defensible adsorption “dose”—accounting for sorbent saturation kinetics and treatment duration—remains undefined [6].

The cost-effectiveness of EBP has not been established. Device costs are substantial and are compounded by the need for additional circuits, anticoagulation, monitoring and nursing time; in the absence of demonstrated benefit, the 2026 SSC panel explicitly weighed these resource requirements against the uncertain effect on mortality [12]. Formal cost-effectiveness analyses, ideally conducted alongside phenotype-enriched trials, are needed before broader adoption can be justified.

## 4. Conclusions

Although EBP has been used in critically ill patients for decades, its role in septic shock remains unproven, an uncertainty driven less by a lack of trials than by their heterogeneity and, above all, by the enrollment of biologically unselected patients. The 2026 SSC guidelines accordingly suggest against the use of blood purification techniques and, specifically, against polymyxin-B hemoperfusion in adults with sepsis or septic shock [12], a position concordant with the 30th ADQI consensus [7] and with both sides of the 2025 PRO–CON debate on hemoadsorption [89,90], and acknowledged even by a recent position statement advocating a personalized approach to hemoadsorption, which concedes that its adoption has rested on pathophysiological rationale rather than on large randomized trials [30]: outside a clinical trial, EBP should not be applied routinely. The identification of a potentially targetable mediator, biomarker or endotoxin burden provides a biological rationale for patient selection in future trials but does not, by itself, establish clinical efficacy. Tigris suggests that phenotype enrichment may reveal a benefit that is diluted in unselected populations, but given its small sample, the imprecision of its primary estimate and the influence of the informative prior, its findings should be regarded as promising evidence requiring independent confirmation rather than as an established clinical benefit, and they cannot be extrapolated to other EBP modalities [16]. The next step is therefore not another broad randomized trial but phenotype-enriched, adaptively designed studies, such as EXCHANGE-2 for plasma exchange [85], using standardized co-interventions, modern ancillary care and endpoints that capture biological as well as vital-status effects [86]. Until such evidence is available, and consistently with the 2026 SSC recommendations [12], EBP is best regarded as an investigational intervention, preferably delivered within clinical trials or prospective registries, rather than as an established rescue therapy or a component of routine sepsis care.

## Figures and Tables

**Figure 1:**
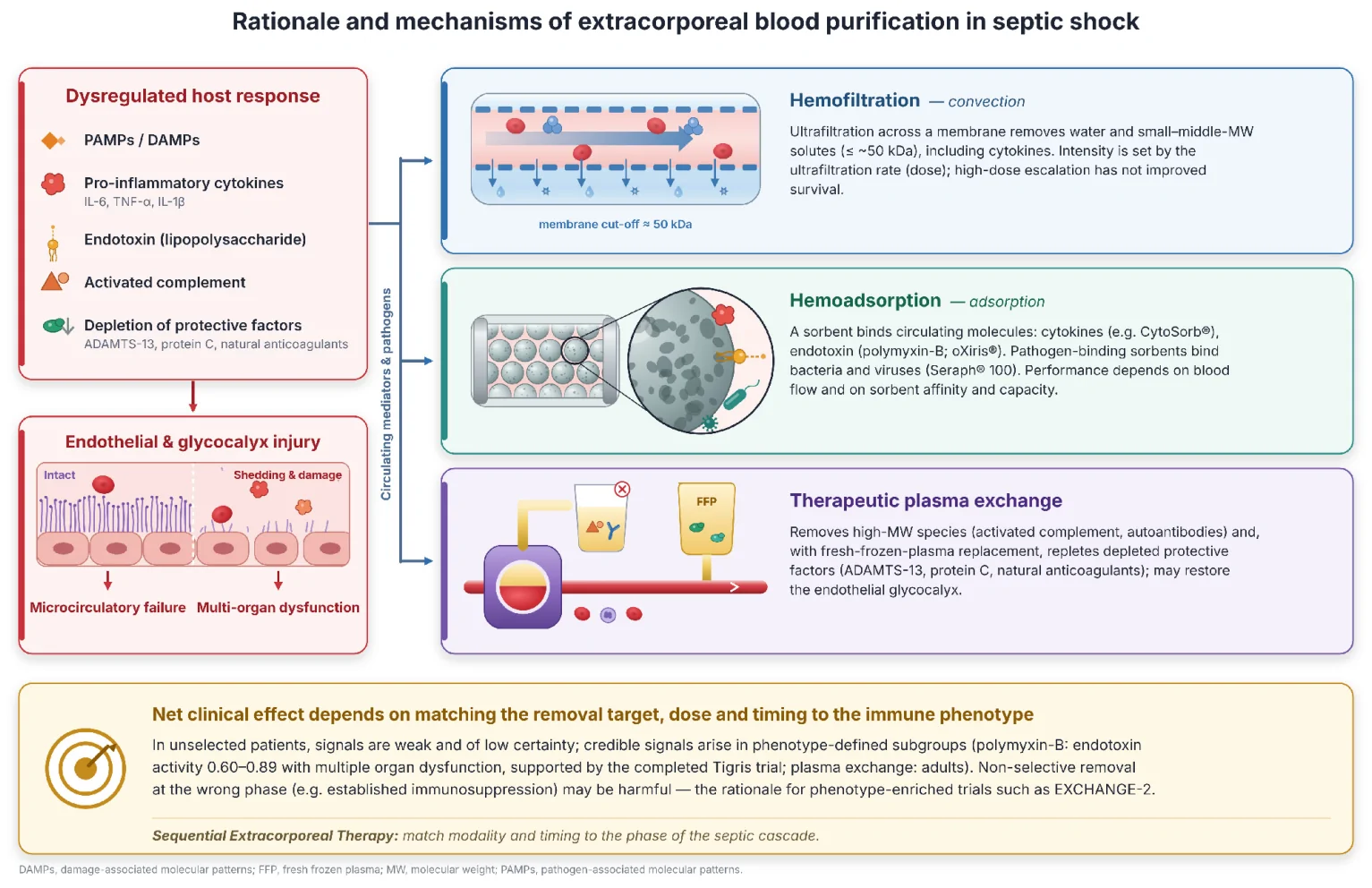
Rationale and mechanisms of extracorporeal blood purification in septic shock. The dysregulated host response to infection releases pathogen- and damage-associated molecular patterns (PAMPs and DAMPs), pro-inflammatory cytokines, endotoxin (lipopolysaccharide) and activated complement, and depletes protective plasma factors (ADAMTS-13, protein C, natural anticoagulants), driving endothelial and glycocalyx injury, microcirculatory failure and multi-organ dysfunction. Three families of extracorporeal techniques act on circulating mediators and pathogens through distinct physical principles: hemofiltration (convective removal of small and middle-molecular-weight solutes), hemoadsorption (sorbent binding of cytokines, endotoxin or whole pathogens) and therapeutic plasma exchange (removal of high-molecular-weight species, with repletion of protective factors when fresh-frozen plasma [FFP] is the replacement fluid). In unselected patients, the signals of benefit are weak and of low certainty; the most promising signals have emerged in phenotype-defined subgroups, largely from exploratory analyses. The net clinical effect therefore depends on matching the removal target, dose and timing to the patient’s immune phenotype. MW, molecular weight.

**Figure 2:**
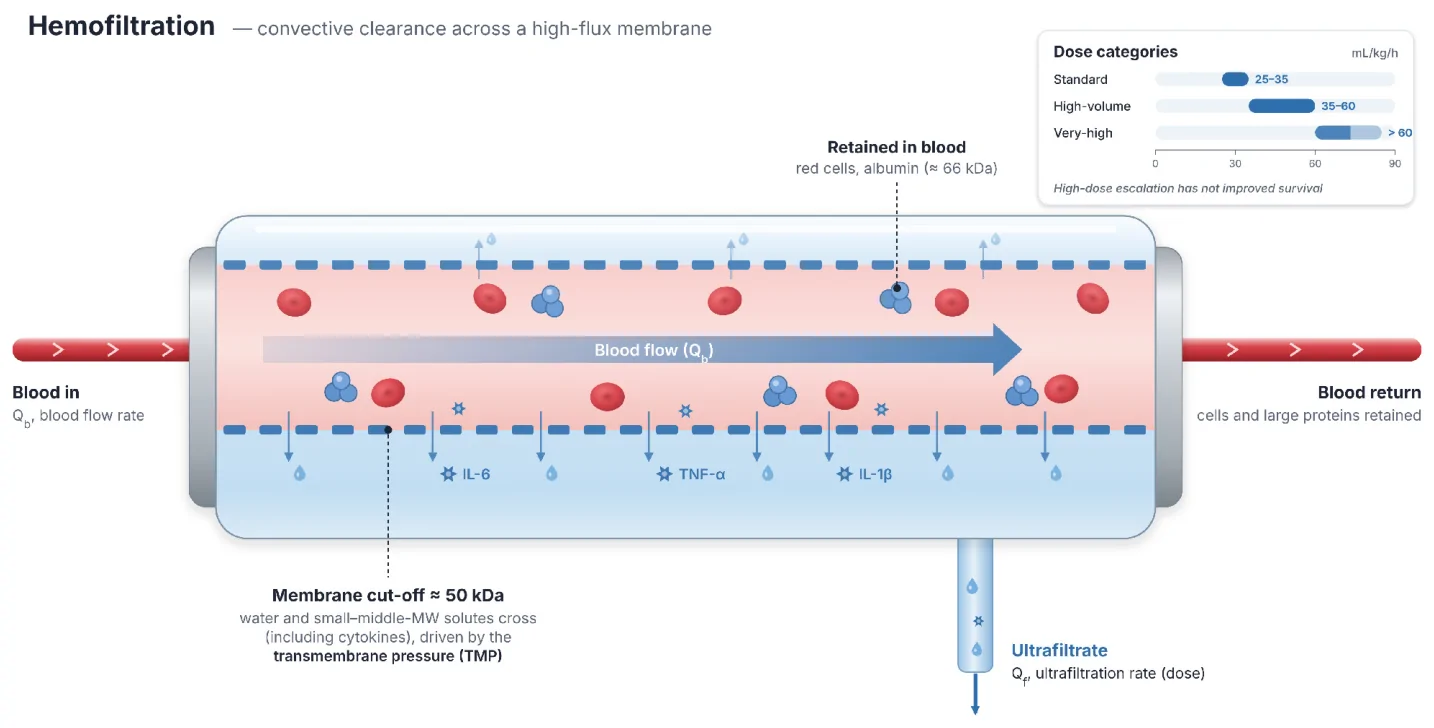
Hemofiltration. Blood flows (Qb) along the hollow fiber of a high-flux membrane; driven by the transmembrane pressure, water and small and middle-molecular-weight solutes, including cytokines (e.g., IL-6, TNF-α, IL-1β), cross the membrane (cut-off ≈50 kDa) by convection and are removed in the ultrafiltrate, whereas cells and large proteins such as albumin (≈66 kDa) are retained. The ultrafiltration rate (Qf) defines the dose; the inset shows the dose categories used in this review (standard 25–35, high-volume 35–60 and very-high-volume >60 mL/kg/h). Escalation of the convective dose has not improved survival.

**Figure 3:**
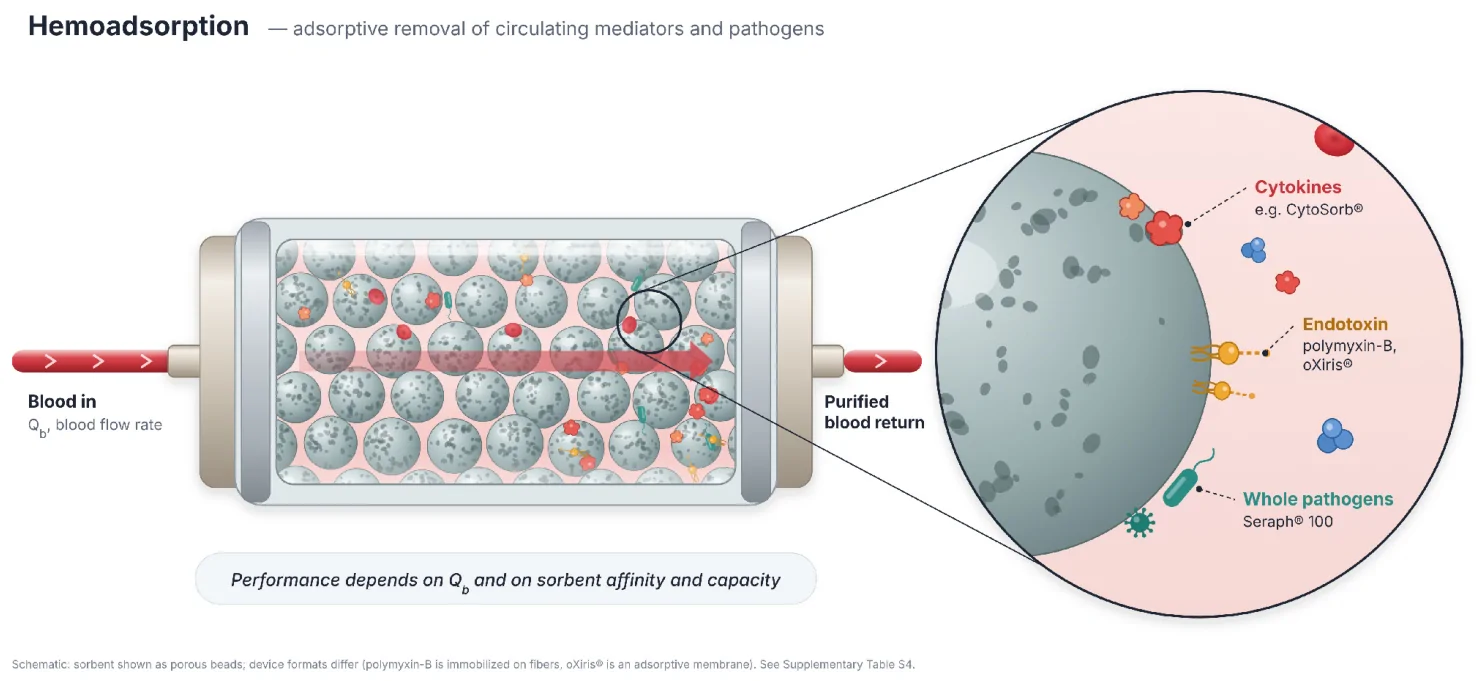
Hemoadsorption. Whole blood (Qb) passes through a cartridge containing a porous sorbent, to which circulating molecules bind. The magnified view shows the main targets with representative devices: cytokines (e.g., CytoSorb^®^), endotoxin (polymyxin-B hemoperfusion, oXiris^®^) and whole pathogens (Seraph^®^ 100). Removal depends on Qb and on the affinity and capacity of the sorbent. The sorbent is shown schematically as porous beads; device formats differ (polymyxin B is immobilized on fibers and oXiris^®^ is an adsorptive membrane; see Supplementary Table S4).

**Figure 4:**
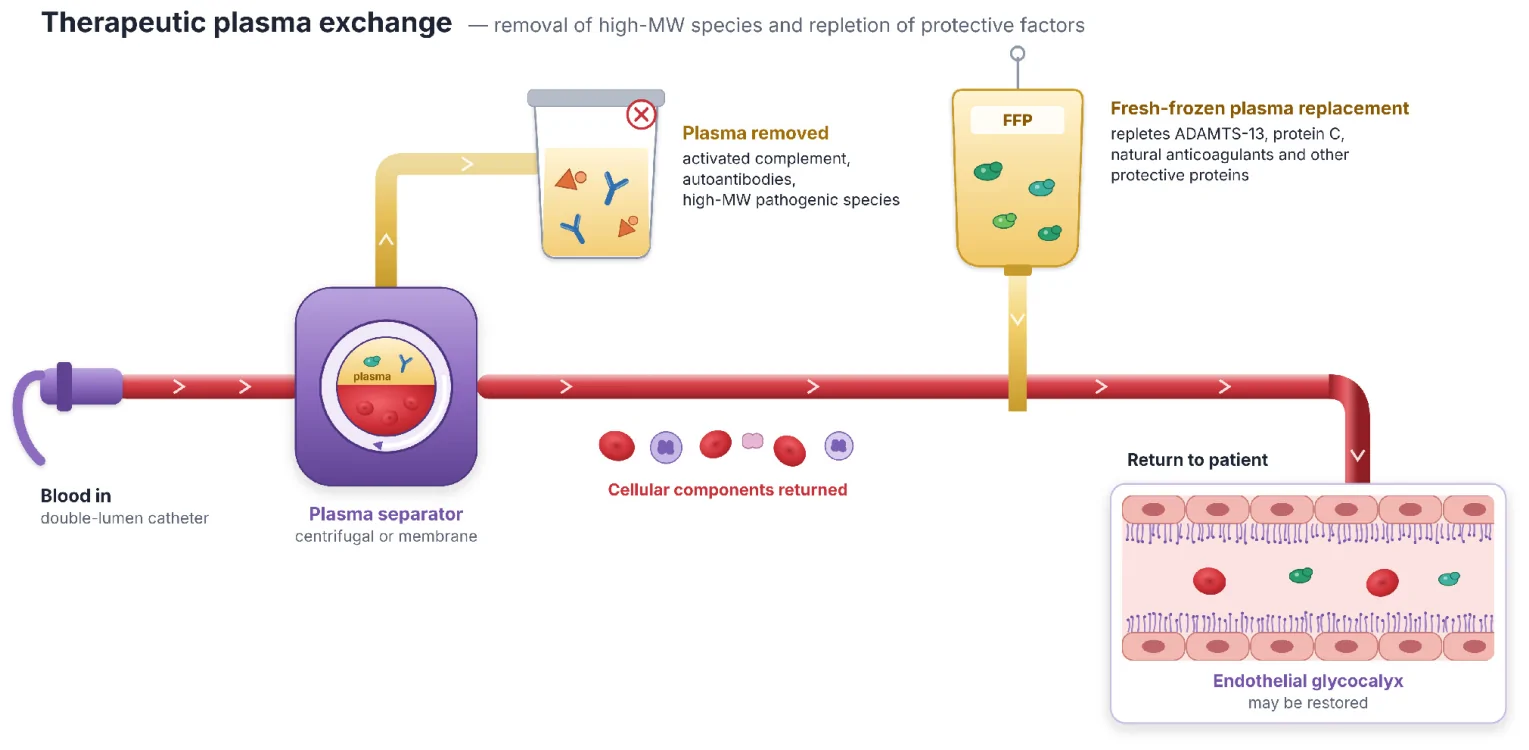
Therapeutic plasma exchange. Blood drawn through a double-lumen catheter is separated by centrifugation or membrane filtration; the removed plasma, containing high-molecular-weight species such as activated complement and autoantibodies, is discarded, and the cellular components are returned to the patient together with the replacement fluid. When fresh-frozen plasma (FFP) is used, it repletes ADAMTS-13, protein C, natural anticoagulants and other protective proteins depleted in sepsis; restoration of the endothelial glycocalyx has also been reported [24].

**Table 1:** Summary of the controlled evidence for extracorporeal blood purification in sepsis/septic shock, by modality, as synthesized by the network meta-analysis of Chen et al. [32] and the 2026 Surviving Sepsis Campaign (SSC) [12].

Technique Group	Modality	Trials, *n* ^1^	Mortality RR (95% CI) vs. Standard Care ^2^	Certainty (GRADE) ^2^	SSC 2026 Pooled Estimate, per Technique Class ^3^	SSC 2026 Recommendation ^4^
**Hemoperfusion/hemoadsorption**	Polymyxin-B hemoperfusion (Toraymyxin^®^)	20	0.70 (0.58–0.86)	Very low	Hemoperfusion/hemadsorption, all devices (41 RCTs): 28–30 d RR 0.83 (0.71–0.98), low; longer-term RR 1.11 (0.94–1.30; 4 RCTs), low	Against (specific rec.), conditional, low
	**CytoSorb^®^**	6	**1.39 (0.97–1.98)**	**Moderate**		Against (general EBP rec.), conditional, very low
	oXiris^®^	1	0.72 (0.29–1.78)	Very low		Against (general EBP rec.), conditional, very low
	HA330 hemoperfusion	2	0.61 (0.35–1.09)	Very low		Against (general EBP rec.), conditional, very low
	Alteco^®^ LPS adsorber	2	0.29 (0.05–1.20) *	Very low		Against (general EBP rec.), conditional, very low
	Immobilized human serum albumin	1	1.12 (0.54–2.35)	Very low		Against (general EBP rec.), conditional, very low
	Selective cytopheretic device (SCD) ^5^	1	1.29 (0.65–1.54) *	Very low		Against (general EBP rec.), conditional, very low
**Hemofiltration (convective)**	Standard-volume CVVH (25–35 mL/kg/h)	7	0.86 (0.61–1.23)	Very low	Hemofiltration (26 RCTs): 28–30 d RR 0.61 (0.61–0.90) †, low (risk of bias, inconsistency); longer-term RR 0.93 (0.78–1.11; 5 RCTs), very low	Not addressed (only high-dose HF is named)
	High-volume CVVH (35–60 mL/kg/h)	2	0.67 (0.41–1.10)	Very low		Against (general EBP rec.), conditional, very low
	Very-high-volume CVVH (>60 mL/kg/h)	6	0.71 (0.43–1.15)	Very low		Against (general EBP rec.), conditional, very low
	Pulse high-volume CVVH	1	0.50 (0.05–5.01)	Very low		Against (general EBP rec.), conditional, very low
**Combined hemoadsorption + hemofiltration (blood-processing)**	HA330 + high-volume CVVH	1	0.58 (0.17–1.93)	Very low	—	Against (general EBP rec.), conditional, very low
	HA330 + pulse high-volume CVVH	1	0.63 (0.20–1.91)	Very low		Against (general EBP rec.), conditional, very low
**Plasma-processing techniques**	Plasma exchange	5	0.61 (0.42–0.91)	Low	Plasmapheresis (4 RCTs): 28–30 d RR 0.64 (0.46–0.89), low	No separate rec.; included in general EBP rec. (against, conditional, very low)
	CPFA	3	1.20 (0.82–1.76)	Very low		Against (general EBP rec.), conditional, very low
	CPFA + standard-volume CVVH	1	0.47 (0.19–1.19)	Very low		Against (general EBP rec.), conditional, very low

Populations: adults with severe infection, sepsis or septic shock (not restricted to septic shock). ^1^ Number of randomized trials of each modality among the 60 included in Chen et al. (Supplementary Table S1); the 16 modalities and counts sum to 60 (33 hemoperfusion/hemoadsorption, 16 hemofiltration, 2 combined, 9 plasma-processing). The polymyxin-B pairwise mortality analysis in Chen et al. was based on 18 of these 20 trials. ^2^ Mortality relative risk (RR, 95% CI) versus standard care and GRADE certainty as reported by Chen et al. (Table S2); the GRADE rating applies to the pooled body of evidence, not to individual trials. * As printed in Table 2 of Chen et al.; these upper confidence limits are not arithmetically consistent with the corresponding point estimates and lower limits and could not be verified against the source data. ^3^ Updated pooled estimates reported by the SSC 2026 panel for each technique class as a whole (all devices within the class), not for individual modalities; no class estimate is reported for combined or CPFA techniques. † Printed as 0.61 (0.61–0.90) in the guideline: the point estimate equals the lower confidence limit, which suggests a typographical error in the source. ^4^ SSC 2026 recommendations for adults with sepsis or septic shock: “we suggest against using blood purification techniques”, including hemoperfusion, high-dose hemofiltration or plasma exchange (general EBP rec.; conditional, very low certainty); “we suggest against using polymyxin B hemoperfusion” (specific rec.; conditional, low certainty). No device-specific recommendation exists for other sorbents or for plasma exchange. Plasma-exchange evidence is additionally informed by Rimmer et al. [31] (adult subgroup RR 0.63, 95% CI 0.42–0.96). ^5^ Leukocyte-processing device, grouped with hemoperfusion as in Chen et al.; neither synthesis includes the phase 3 Tigris trial of polymyxin-B hemoadsorption in endotoxic septic shock (endotoxin activity 0.60–0.89), published in 2026 [16], or the 2026 RCTs of CytoSorb^®^ [25] and HA330 [26] (see text). Hemofiltration doses (ultrafiltration rate, Qf) are expressed in mL/kg/h, with the categories defined in the text. EBP, extracorporeal blood purification; CPFA, coupled plasma filtration and adsorption; CVVH, continuous veno-venous hemofiltration; HF, hemofiltration; SCD, selective cytopheretic device. CytoSorb^®^ (bold) is the only modality graded at moderate certainty and the only one whose point estimate favors harm.

## Data Availability

No new data were created or analyzed in this study; all data discussed are available in the cited publications.

## Supplementary Materials

The following supporting information can be downloaded at: https://www.mdpi.com/article/10.3390/jcm15218619/s1, Supplementary Table S1: characteristics of the randomized controlled trials included in the source syntheses; Supplementary Table S2: search strategy and update; Supplementary Table S3: extracorporeal blood purification devices in clinical use or under evaluation; Supplementary Table S4: characteristics of available hemoadsorption cartridges.
