# Supplementary material for: Extracorporeal Blood Purification Techniques in Septic Shock: Operating Principles, Supporting Evidence and Clinical Applications
Source: J Clin Med. 2026 Nov 5;15(21):8619. doi: 10.3390/jcm15218619 (PMC13764423; doi:10.3390/jcm15218619)
Supplement: Supplementary file 1 [file jcm-15-08619-s001.zip › jcm-4597849-supplementary.pdf]

Supplementary Table S1: characteristics of the randomized controlled trials included in the source syntheses

Technique group	Device	Trials (Chen)	Mortality RR (95% CI) vs standard — Chen 2023	GRADE certainty	P-score (rank)
Hemoperfusion / hemoadsorption	Toraymixin®	20	0.70 (0.58-0.86)	Very low	0.58
	CytoSorb®	6	1.39 (0.97-1.98)	Moderate	0.10
	oXiris®	1	0.72 (0.29-1.78)	Very low	0.54
	HA330®	2	0.61 (0.35-1.09)	Very low	0.66
	Alteco LPS® a	2	0.29 (0.05-1.60)	Very low	0.83
	iHSA®	1	1.12 (0.54-2.35)	Very low	0.26
Ultrafiltration	Standard-volume CVVH	(multiple)	0.86 (0.61-1.23)	Very low	0.40
	High-volume CVVH		0.67 (0.41-1.10)	Very low	0.61
	Very high-volume CVVH		0.71 (0.43-1.15)	Very low	0.57
	Pulse high-volume CVVH		0.50 (0.05-5.01)	Very low	0.63
	CPFA	3	1.20 (0.82-1.76)	Very low	0.18
	CPFA + standard-volume CVVH	1	0.47 (0.19-1.19)	Very low	0.77
	HA330 + high-volume CVVH	1	0.63 (0.20-1.91)	Very low/mod	0.60
	HA330 + pulse high-volume CVVH	1	0.58 (0.17-1.93)	Very low	0.64
Plasma exchange	Plasma exchange devices	5 (Chen) / 4 (Rimmer)	0.61 (0.42-0.91)	Low	0.67

Legend: CVVH: continuous veno-venous hemofiltration

Supplementary Table S2: search strategy and update.

Line	Search string	Concept
**#1**	"Shock, Septic"[Mesh] OR "Sepsis"[Mesh] OR septic shock[tiab] OR sepsis[tiab] OR septicemia[tiab] OR septicaemia[tiab] OR "bloodstream infection"[tiab] OR bacteremia[tiab] OR bacteraemia[tiab] OR "severe sepsis"[tiab]	Sepsis and septic shock terms
**#2**	"Hemofiltration"[Mesh] OR "Hemoperfusion"[Mesh] OR "Renal Replacement Therapy"[Mesh] OR "Plasma Exchange"[Mesh] OR "Hemodiafiltration"[Mesh] OR "Blood purification"[tiab] OR "extracorporeal blood purification"[tiab] OR hemofiltrat*[tiab] OR haemofiltrat*[tiab] OR hemoperfus*[tiab] OR haemoperfus*[tiab] OR hemadsorpt*[tiab] OR haemadsorpt*[tiab] OR hemoadsorpt*[tiab] OR haemoadsorpt*[tiab] OR "blood adsorpt*"[tiab] OR "adsorption therapy"[tiab] OR "hemodiafiltration"[tiab] OR "hemodiafiltration"[tiab] OR "cytosorb"[tiab] OR "oXiris"[tiab] OR "Seraph 100"[tiab] OR "Seraph100"[tiab] OR "Toraymyxin"[tiab] OR "hemopurifier"[tiab] OR "polymyxin B hemoperfusion"[tiab] OR "cytokine adsorption"[tiab] OR "cytokine removal"[tiab] OR "coupled plasma filtration and adsorption"[tiab] OR "CPFA"[tiab] OR plasmapheresis[tiab] OR "plasma exchange"[tiab] OR "continuous renal replacement therapy"[tiab] OR CRRT[tiab]	Blood purification terms
**#3**	#1 AND #2	Combine infection and blood purification terms

**#4**	#3 AND ("2022/01/01"[Date - Publication] : "2025/08/05"[Date - Publication])	Limit to publications between Jan 1 2022 and Aug 5 2025

Supplementary Table S3: extracorporeal blood purification devices in clinical use or under evaluation

Denomination	Mechanism of action	Modality	Manufacturer	Recommended Duration (hours* days)
Hemofiltration (at different QF: see chapter below, HF)	Convective Removal of mediators	CRRT	Different	24-72 hours
Alteco®	Adsorption of LPS	Stand alone	Alteco Medical	2-6 * 2
oXiris®	Combined HA of LPS and convective removal of mediators	CRRT	Baxter	n.d.
Toraymixin®	HA of LPS on polymyxin B columns	Stand alone	Toray	2 * 2
Matisse®	HA of LPS/DAMPS/PAMPS on albumin microbeads	CRRT	Fresenius	3-4
Seraph 100®	HA of pathogens/DAMPS/PAMPS on heparan sulphate microbeads	Stand alone	Extera Medical	4 (extension to 24 pending)
Hemopurifier®	HA of pathogens to synthetic agglutinin.	Stand alone / CRRT	Aethlon Medica	4

Garnet®	HA of pathogens on mannose-binding lectin	Stand alone / CRRT	BOA Biomedical	Not determined
Cytosorb®	HA of mediators on polystyrene microbeads	Stand alone / CRRT	Cytosorbents	24
HA 330®	HA of mediators on resin macroporous column	Stand alone / CRRT	Jafron	2*3 days
Therapeutic Plasma Exchange	Removal and substitution of 30-40 ml/kg of plasma volume	Stand alone	Different	As needed

Legend: CRRT, continuous renal replacement therapy.

Legend: CRRT: continuous renal replacement therapy.

Supplementary **Table S4: Characteristics of available hemoadsorption cartridges**

Cartridge	Sorbent	3D Arrangement	Surface (m²)
Cytosorb®	Polystyrene divinylbenzene copolymer	Microbeads	45
Alteco®	Peptide with high affinity for LPS	Micro discs	4
oXiris®	Mediators/DAMPS/PAMPS: LPS: polycationic polyethyleneimine (PEI)	Hollow fibers	1,5
Toraymixin®	Polymyxin-B	Hollow fibers	500

Matisse®	Immobilized human albumin	Microbeads	n.a.
HA330®	neutral microporous resin and collodion coating	Microbeads	10
Seraph 100®	Ultra-high MW coated with end-pint attached heparin	Microbeads	40
Hemopurifier®	Galanthus nivalis agglutinin	Hollow fibers	n.a.
Garnet®	Fc-mannose-binding lectin	Hollow fibers	n.a.
Efferon LPS®	Unspecified	Microbeads	n.a.

Legend: DAMPS: damage-associated molecular patterns; PAMPS: pathogen-associated molecular patterns; LPS: lipopolysaccharide.
